# Pollination of *Cambessedesia wurdackii* in Brazilian Campo Rupestre Vegetation, with Special Reference to Crepuscular Bees

**DOI:** 10.1673/031.011.9701

**Published:** 2011-07-27

**Authors:** Emanuella Lopes Franco, Miriam Gimenes

**Affiliations:** Departamento de Ciências Biológicas, Universidade Estadual de Feira de Santana, Avenida Transnordestina, S/N, Bairro Novo Horizonte, 44036-900 Feira de Santana — BA, Brasil

**Keywords:** montane savanna, Colletidae, Halictidae, *Megalopta*, *Ptiloglossa*

## Abstract

*Cambessedesia wurdackii* Martins (Myrtales: Melastomataceae) is presumably endemic to the Chapada Diamantina, Bahia State, Brazil. A majority of the species of this family are pollinated by diurnal bees that buzz the floral anthers to collect pollen. The present work examined the interactions between *C. wurdackii* and visiting bees, focusing on temporal, morphological, and behavioral features, especially in regards to the crepuscular bees *Megalopta sodalis* (Vachal) (Hymenoptera: Halictidae) and *Ptiloglossa off. dubia* Moure (Hymenoptera: Colletidae). The study was undertaken in an area of campo rupestre montane savanna vegetation located in the Chapada Diamantina Mountains of Bahia State, Brazil, between August/2007 and July/2008. Flowering in *C. wurdackii* occurred from April through July, with a peak in May. A total of 592 visits by diurnal and crepuscular bees to the flowers of *C. wurdackii* were recorded, with a majority of the visits made by *M. sodalis* and *P. dubia* (92%) near sunrise and sunset. The anthers of *C. wurdackii* are arranged in two tiers, which favors cross pollination. The morphological, temporal and behavioral characteristics of *M. sodalis* and *P. dubia* indicated that they were potential pollinators of *C. wurdackii*, in spite of the fact that the colorful and showy flowers of this species are more typical of a diurnal melittophilous pollination syndrome.

## Introduction

The botanical family Melastomataceae is well represented in tropical and subtropical regions of the Americas (Renner et al. 2001). It is the sixth largest Angiosperm family in Brazil in terms of the number of species, with representatives being found in many diverse habitats, such as the Atlantic Forest (Melo et al. 1999), Amazonian Forest (Renner 1987), *Cerrado* (savanna) (Renner 1990), dune and beach-front areas (Alves-dos-Santos 1999), and especially in areas of campo rupestre (high-altitude open, rocky areas) (Romero and Martins 2002). The genus *Cambessedesia* occurs in Brazil and many species occur in campo rupestre vegetation (Martins 1984), while others are found in areas of *cerrado* (Joly 1987). *Cambessedesia wurdackii* Martins (Myrtales: Melastomataceae) is geographically restricted to Bahia State in the Chapada Diamantina Mountains (Martins 1984).

Characteristic of the family Melastomataceae, species of *Cambessedesia* have flowers with rich colors and poricidal anthers. These flowers are classified as melittophilous and are visited almost exclusively by bees that buzz the anthers to release their pollen (Vogel 1978; Faegri and Van Der Pijl 1979; Renner 1989). In a study of pollination and the reproductive systems of *Cambessedesia hilariana* in areas of campo rupestre and *cerrado* vegetation in São Paulo State, large, diurnal bees of the genera *Centris, Bombus*, and *Xylocopa* were the principal pollinators (Fracasso and Sazima 2004).

Plants with floral morphologies similar to those seen in the genus *Cambessedesia* are melittophilous, and this syndrome is generally associated with the diurnal foraging of certain bees. Nonetheless, some bee species belonging to four of the seven bee families (Colletidae, Andrenidae, Halictidae and Apidae) have independently adopted the habit of foraging when there is very little daylight, concentrating their activities during the late evening hours or at daybreak (Hopkins et al. 2000; Wcislo et al. 2004; Warrant 2008). The evolution of this activity pattern is probably related to the exploitation of richer floral resources and avoidance of competitors, predators and parasites with diurnal habits (Wicslo et al. 2004; Kelber et al. 2006).

In general, bee species that have crepuscular habits, such as species of the genus *Ptiloglossa* (Hymenoptera: Colletidae) (Roberts 1971) and *Megalopta* (Halictidae) (Janzen 1968; Kelber et al. 2006), forage just before daybreak and just after sunset. In a study on the relationship between the sizes of the ocellus of some crepuscular bee species and their foraging activity, it was noted that ocellus size is not directly proportional to head size, principally among crepuscular and nocturnal species in which ocellus size tended to be larger (Kerfoot 1967).

The knowledge of the floral resources utilized by nocturnal and crepuscular bees is still incipient and quite limited. Researchers that have analyzed pollen samples from nests of species within the genus *Megalopta* in tropical regions have identified more than 40 plant species with diurnal and/or nocturnal anthesis belonging to the families Bombacaceae, Anacardiaceae, Guttiferae, and Melastomataceae (Roulston 1998; Wicslo et al. 2004). *Megalopta centralis* visited the flowers of *Solanum* spp. (Solanaceae) and *Calathea insignis* Petersen (Marantaceae) in Costa Rica (Janzen 1968). *Megalopta* spp. were considered as pollinators of *Parkia* *velutina* Benoist in the Amazon region (Brazil) (Hopkins et al. 2000), and species of the genus *Ptiloglossa* were recorded visiting flowers of Solanaceae (Linsley and Cazier 1970), Melastomataceae (Roberts 1971), and *Ipomoea* (Convolvulaceae) (Schlising 1970).

The purpose of this study was to describe interactions between *C. wurdackii* and visiting bees, focusing on the morphological, behavioral and temporal aspects of the visits of the bees in relation to temperature, relative humidity, light intensity and daily light/dark cycle; and especially in terms of the crepuscular bee species *Megalopta sodalis* Vachal (Hymenoptera: Halictidae) and *Ptiloglossa* aff. *dubia* Moure (Colletidae).

## Methods

### Study area

The present study was undertaken in an area of campo rupestre vegetation inside the Mucugê Municipal Park (12° 59′ 18.5″ S × 41° 20′ 27.8″W; at 950 m altitude), the headquarters of the “Sempre Viva” Project. This vegetation is characterized principally by a herbaceous-shrub physiognomy with a high degree of endemism. The region has extensive areas of exposed rock and a low water-retention capacity. The plant families showing the greatest species richness in the *campos rupestre* vegetation of the Chapada Diamantina Mountain Range are Orchidaceae, Poaceae, Asteraceae, Velloziaceae, Bromeliaceae, and Melastomataceae (Harley 1995; Conceição et al. 2007a, 2007b). The local climate is semi-humid, with irregular annual rainfall between 600 and 1500 mm. Average temperatures vary between 13° C in the dry season (April to September) and 30° C in the rainy season (October to March), although large variation may occur between years (Stradmann et al. 1998).

### Environmental and meteorological data

Data on rainfall, relative humidity, and monthly average temperature from August 2007 to July 2008 were obtained from the meteorological station at the Municipal Park. Sunrise and sunset times were obtained from the Brazilian National Observatory (http://euler.on.br/ephemeris/index.php). Temperature, relative humidity, and light intensity data were collected in the field during the observations of floral visitors, with at least one measurement being made every hour. Light intensity was measured using a digital luximeter (Lutron LX-107, www.lutron.com) at a distance of 100 cm from the soil.

### Floral Biology

The individuals of *C. wurdackii* that were studied were sparsely distributed woody shrubs approximately 50 cm tall growing on rocky and inclined river margins. To evaluate the flowering phenology of *C. wurdackii*, 11 individual plants were marked and monitored from August 2007 through July 2008. Observations of floral biology and floral visitors were performed on three days during June 2007, late May – early June, and late June – early July 2008 (months with great numbers of flowers) (total observation period of 9 days).

The timing of floral opening and the durations of the flowers were followed in 10 flowers on different plants. The stigmas were cut off and submerged in 10% hydrogen peroxide solution. Stigma receptivity was evaluated based on the intensity of effervescence (Zeisler 1933 in Dafni and Maués 1998) on three pre-anthesis buds (at a time close to, but still before the full opening of the flowers) and on three flowers at different phases of anthesis (3 hours, 27 hours, 59 hours, and 75 hours after opening). To determine the presence of osmophors, ten flowers were submerged in a solution of 1:10.000 neutral red: tap water for 10 min. Flowers were then rinsed with tap water (Vogel 1963 in Ormond et al. 1981). To determine the presence of pigments that absorb in the ultraviolet spectrum, ten flowers were placed in a glass container and exposed to ammonium hydroxide vapor. (Scogin et al 1977).

Ten flowers from different plants were chosen to measure the corolla diameter, anther length, style length , the width of the upper anther clusters, and the distance between the upper anther cluster and the stigma.

### Floral visitors

In order to observe the activities of the floral visitors, a 100 m^2^ plot was selected in the study area during each field trip in a locality with large number of flowers. This plot was used to make behavioral observations of the bees and to record their visiting activities during three days each month (04:30 – 19:30) (before dawn to after sunset). The total number of visits of each bee species was noted during two intervals of 15 minutes during each hour of observation. The bee visit count was based on the number of times each bee buzzed the flowers for pollen collection. In addition to these regular observations, three days of observations were made throughout the night (18:00 – 06:00) in June 2007 in order to determine if the flowers of *C. wurdackii* were visited during these hours.

Observations of floral visitor behavior were made during each hour interval in the field, mainly at the moment when a bee arrived near each flower. The way the insect's body made contact with the stigma and its behavior while collecting the floral resource were noted. Photographs and digital films were made of the floral visits to aid in these analyses.

To analyze the morphological characteristics of each bees species, 3–10 individuals were collected to measure their body length (between the median ocellus and the end of the abdomen) and intertegular width (the distance between wing bases). Additional measurements were made of the width of the median ocellus and the width of the head, following Kerfoot (1967). The ratio of median ocellus width to head width was related to the light intensity at the time of each species' peak of visitation.

The collected bees were deposited in the Prof. Johann Becker Entomological Collection of the Museu de Zoologia da UEFS, and vouchers of *C. wurdackii* were deposited in the Herbarium of the Universidade Estadual de Feira de Santana (HUEFS).

### Data analysis

The flowering pattern of this species was classified using the scale developed by Newstron et al. (1994) according to its frequency and duration.

The frequency of bee visits to the flowers was calculated based on the percentage of the total number of visits to a species in relation to the total number of visits observed.

Spearman's correlation was used (at p < 0.05) to analyze the influence of climatic factors on flowering, as well as the influence of climatic factors during the day on the numbers of bee visits.

## Results

**Figure 1.**
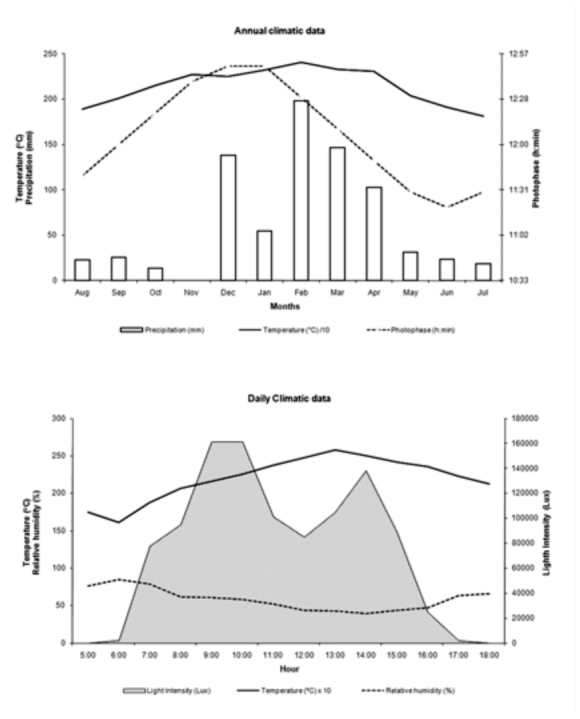
Climate data throughout the year (average temperature, average relative humidity, total precipitation and duration of photophase) (top) and during the days of active observations in the months of May 2008 and June 2008 (average temperature, relative humidity, maximum light intensity) (bottom) in an area of campo rupestre vegetation in Mucugê, Bahia State, Brazil. High quality figures are available online.

Flowering in *C. wurdackii* occurred from April to July, with a total of 150 flowers observed in April, 1062 in May (flowering peak), 185 in June, and only 1 in July. The flowering phase coincided with diminishing values of: the photophase (total period of available light during the day), precipitation, and the average temperature (Figure 1). Of the variables analyzed, only flowering and the average values of the photophase (rs = -0.75) demonstrated a significant negative correlation.

The flowers of *C. wurdackii* are of the open type - pentamerous and zygomorphic - with green sepals and oval petals with two showy colors: yellow at the basal quarter and orange colored above, reflecting iridescently in the sunlight. Pollen is the only floral reward offered to insect visitors. The corolla tube had a diameter of 18 ± 0.92 mm. The style was 10.9 ± 0.57 mm long. The stamens were free and didynamous, each being composed of a filament and an elongated yellow anther that terminated in an apical pore. The anthers were arranged in two clusters: an inferior group formed by three large anthers (mean ± SD = 4.4 ± 0.5 mm of length) that were grouped together with the style and stigma and another upper group formed by seven smaller anthers (3.2 ± 0.55 mm of length), totaling 10 anthers. The flowers had a sweet smell that was most intense in the afternoon and evening. This odor probably arises from osmophores located on the corolla and on the tips of the anthers (as indicated by treatment with neutral red). Tests with ammonium hydroxide vapor did not indicate the presence of pigments that reflect ultraviolet light.

Pre-anthesis initiated about 24 hours before the flowers began to open and was characterized by the elongation of the style and the exposition of the stigma beyond (and therefore outside of) the floral bud. The petals gradually began to open at approximately 01:00, while the anthers remain doubled over in the bud with their pores facing the base of the filaments. As the petals continued to open (about 2 hours after initiating anthesis) the anthers were presented in 2 clusters, giving the flower a strongly zygomorphic symmetry. The stigma was now located beyond the anther pores, characterizing herkogamy. Anthesis terminated at about 05:00 when the petals were totally extended. Twenty-four hours after floral opening the petals gradually acquired a pallid aspect and finally abscised after 72 to 80 hours. The stigma was receptive for the entire period from pre-anthesis to petal fall.

A total of 582 visits were recorded by bees belonging to three families and seven species (Table 1). The present study represents the first report for Bahia State of *P. dubia* (67% of the total number of visits) and *M. sodalis* (25.3%), and these species were observed to be the most frequent visitors to *C. wurdackii*. The other species collectively made only 7.7% of the total visits. *P. dubia, M. sodalis* and *Euglossa* sp. visited *C. wurdackii* flowers during all of the observation months (Table 1). The greatest number of floral visits occurred at the end of June and the beginning of July 2008 (92% of the total number of visits), 5% of the visits occurred at the end of May and beginning of June 2008, and 2% of the visits occurred in June 2007.

**Table 1.**
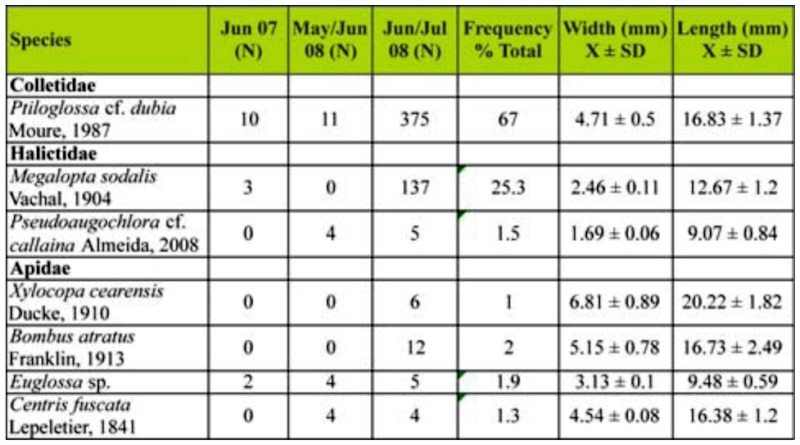
Bees visitors to flowers of *Cambessedesia wurdackii* in an area of campo rupestre vegetation in Mucugê, Bahia State, Brazil. N = number of visits; width (intertegular distance), and lengh (distance between the median ocellus and the end of the abdomen) of the bees.

**Figure 2.**
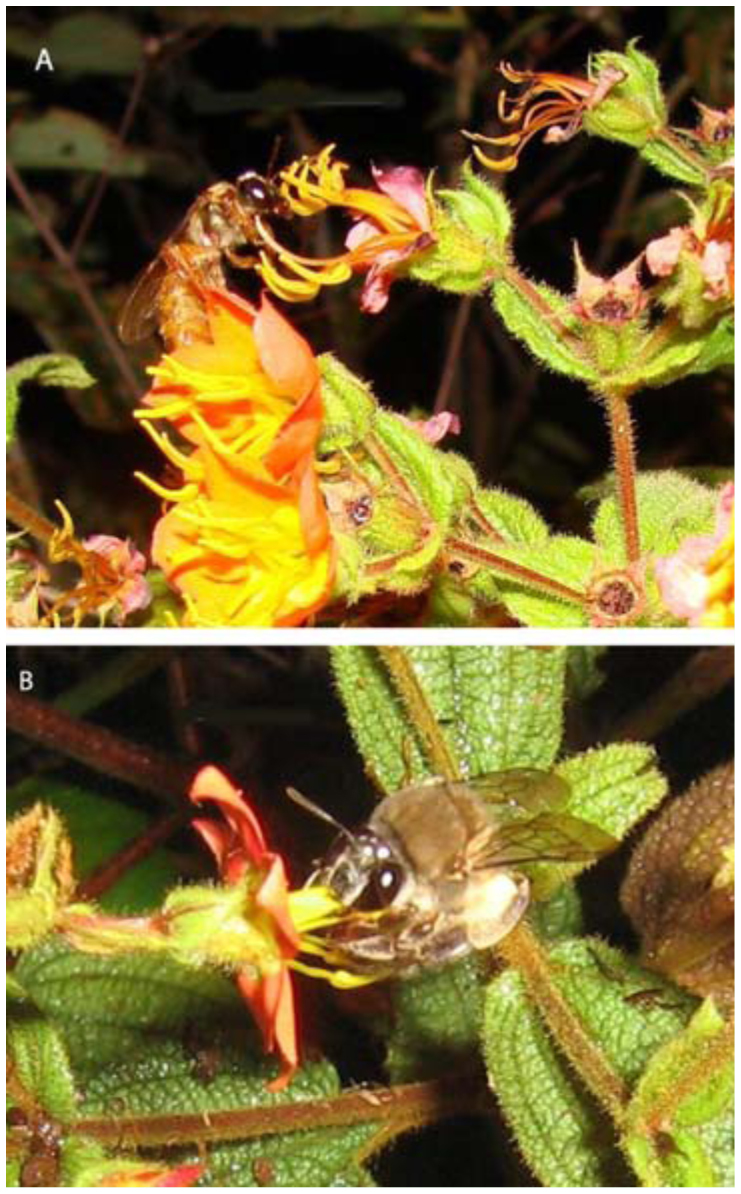
*Megalopta sodalis* (A) and *Ptiloglossa* aff. *dubia* (B) visiting flowers of *Cambessedesia wurdackii* in an area of campo rupestre vegetation in Mucugê, Bahia State, Brazil. High quality figures are available online.

In spite of the fact that all of the bee species visiting *C. wurdackii* flowers buzzed their anthers, no species was able to embrace both the upper and lower clusters of anthers at the same time. A bee would generally embrace only the upper group of anthers (which served as their landing platform), although these insects would occasionally also buzz the lower anther group. When a bee buzzed the upper anthers, the entire flower would vibrate and the pollen would be liberated from both the upper and lower anthers. The pollen liberated from the upper anthers was generally deposited on the ventral portion of a bee's thorax and later transferred to the scopae or corbiculae. The pollen from the lower anthers was generally deposited on the posterior portion of the abdomen of the largest bee visitors where it could come in contact with floral stigmas during subsequent floral visits, and this pollen was apparently not actively transferred to the scopae by those bees (Figure 2).

The average widths of *C. fuscata*, *P. dubia, B. atratus*, and *X. cearensis* were approximately the same size as the average width of the group of upper anthers (mean ± SD = 5.0 ± 0.6 mm), while the remaining bee species were thinner (Table 1). Despite differences in width, the only species that was not able to embrace all of the upper anthers during their floral visits was *P*. cf. *callaina*. The distance between the upper group of anthers and the stigma (9.0 ± 1.35 mm) was slightly less than the average length of the bees (Table 1). However, the bees curved their abdomens while visiting the flowers, thus diminishing their overall effective length; the smallest bees (*P*. cf. *callaina* and *Euglossa* sp.) therefore did not touch the stigma during their visits.

*M. sodalis* generally fly slowly and then amble over the inflorescences, touching the stigma of the flower. This bee generally remained for approximately 20 seconds on each flower. They tended to spend more time on the flower than the other species of bee visitors. *P. dubia, B. atratus*, and *X. cearensis* all made rapid visits, remaining for about 3 seconds on each flower.

**Figure 3.**
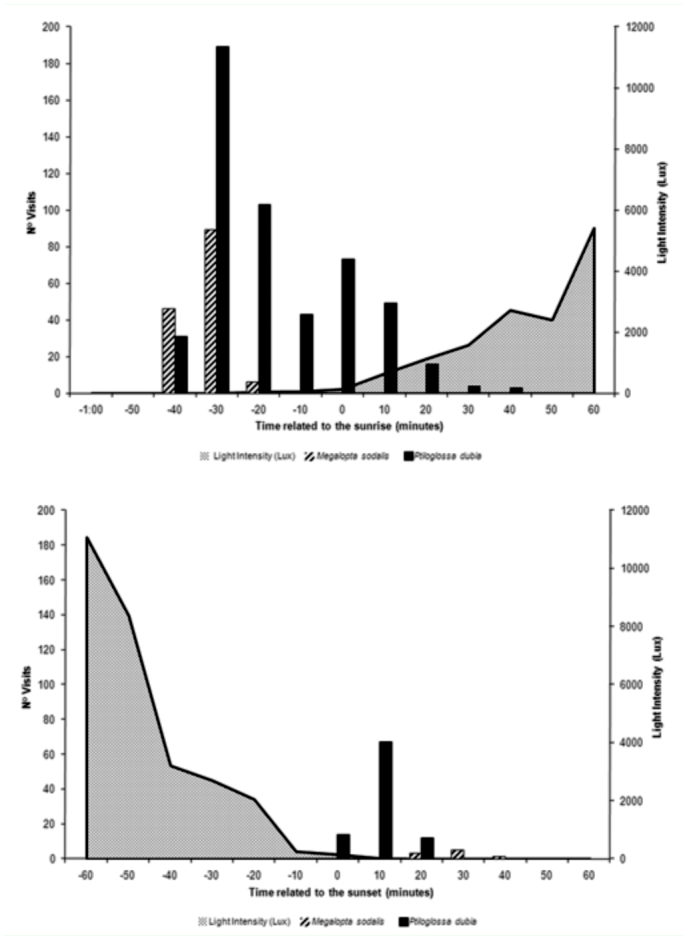
Number of visits of *Megalopta sodalis* and *Ptiloglossa* aff. *dubia* to *Cambessedesia wurdackii* flowers in relation to the time of sunrise (top) and sunset (bottom) (at time = 0) during the months of May 2008 and June 2008, and the corresponding light intensity values in an area of campo rupestre vegetation in Mucugê, Bahia State, Brazil. High quality figures are available online.

*M. sodalis* and *P. dubia* had crepuscular and bimodal activity patterns, with one activity peak in the early morning and another in the late evening (Figure 3). Floral visits by *M. sodalis* and *P. dubia* began at 05:20 (about 40 minutes before sunrise). *M. sodalis* terminated its visits at 05:35, and *P. dubia* at 06:40 (Figure 3). The greatest numbers of visits during the early morning hours occurred between 5:20 and 5:30 for *M. sodalis*, and for *P. dubia*, the greatest numbers of visits occurred between 5:30 and 5:40, with an average temperature near 16.0° C. Both of these bee species visited flowers of *C. wurdackii* around sunset, but visits were less frequently than at sunrise: at 17:40 – 18:00 for *M. sodalis* (N = 9), and at 17:20 – 17:40 for *P. dubia*. The greatest numbers of visits by *P. dubia* during the late evening occurred at 17:30 (N = 67), with an average temperature near 21.0° C. During the entire activity phase of visits by *M. sodalis* the light intensity varied from < 1 lux to 47 lux and by *P. dubia* varied from < 1 lux to 2720 lux (Figure 3).

The Spearman correlation values relating the numbers of visits of each of the bee species to *C. wurdackii* flowers to the microclimatic data (collected during each day) were significant only for the visits of *P. dubia and M. sodalis*. The correlation was positive for the relative humidity and negative for temperature and light intensity, with the greatest correlation values being seen for the latter variables (Table 2).

**Figure 4.**
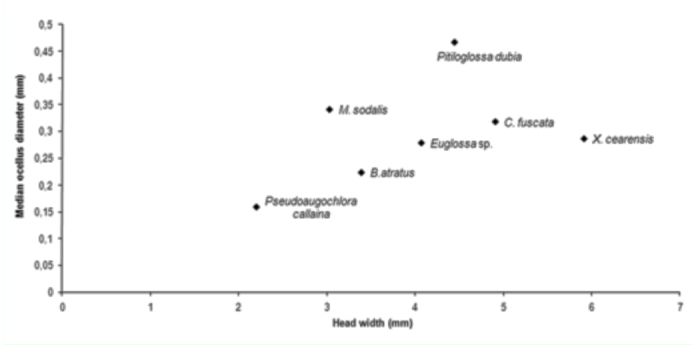
Relationship between the diameter of the median ocellus (mm) and head width (mm) of the bees visiting *Cambessedesia wurdackii* flowers in an area of campo rupestre vegetation in Mucugê, Bahia State, Brazil. High quality figures are available online.

**Figure 5.**
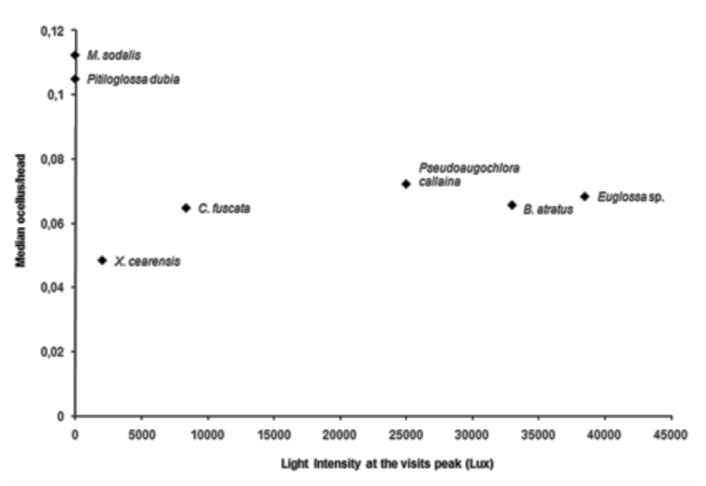
Relationship between the ratio of the diameter of the median ocellus to head width and light intensity (Lux) during the period of peak bee visits to *Cambessedesia wurdackii* flowers in an area of campo rupestre vegetation in Mucugê, Bahia State, Brazil. High quality figures are available online.

The analysis relating the sizes of the median ocellus and the head demonstrated significant differences between bee species with crepuscular and diurnal habits (Figure 4). The nocturnal and crepuscular species (*M. sodalis* and *P. dubia*) had the highest ratios between ocellus size and head width, thus having the largest ocelli even though they did not have the widest heads (Figure 4). The ratios between ocellus size and head width when compared with the light intensity values at the time of peak visitation (Figure 5) indicated that the bee species with proportionally larger ocelli visited *C. wurdackii* flowers during times of lower light intensity than did bee species with proportionally smaller ocelli.

## Discussion

The flowering of *C. wurdackii* in the area of campo rupestre examined was of intermediate duration with a single flowering peak in May (according to the classification of Newstron et al. 1994). In a study of flowering of *C. hilariana* in an area of rupiculous and *cerrado* (savanna) vegetation at 880 m altitude in São Paulo State (Brazil), Fracasso and Sazima (2004) noted that the flowering pattern of *C. hilariana* is long and annual, with flowering from September to July and a peak from October to December. According to this study *C. hilariana* provides abundant resources for bee species during a large part of the year, a situation that was not observed with *C. wurdackii* in the present study.

The flowering activity of *C. wurdackii* and the general morphology of their flowers are consistent with the majority of the *Cambessedesia* species (Martins 1984). *C. wurdackii* flowers, however, emit a sweet aroma, while Martins (1984) noted that the flowers of the genus *Cambessedesia* are odorless. An acrid-saponaceous odor was also reported for flowers of *C. hilariana* (Fracasso and Sazima 2004).

**Table 2.**
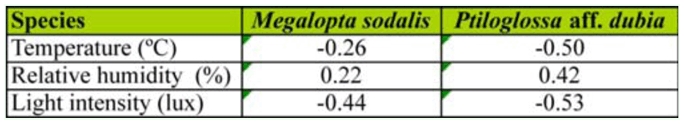
Values of the Spearman correlation (p<0.05) relating climatic variables measured during the day (temperature, relative humidity, and light intensity) with the numbers of visits of *Ptiloglossa* aff. *dubia* and *Megalopta sodalis* to flowers of *Cambessedesia wurdackii* in an area of campo rupestre vegetation in Mucugê, Bahia State, Brazil.

Both the time of day when the flowers of *C. wurdackii* first open and the placement of the floral verticils during anthesis represent a pattern quite different from that described for *C. hilariana* (Fracasso and Sazima 2004). At the initiation of anthesis in *C. hilariana* the flowers demonstrated actinomorphic symmetry, which only changed approximately three hours later with the shifting of the position of the anthers, style and stigma into a single group in the lower part of the flower (Fracasso and Sazima 2004). This pattern of placement of the floral whorls occurs in much the same manner in other species of Melastomataceae (Melo and Machado 1996). In *C. wurdackii*, however, the anthers form two clusters (an upper and a lower) when floral opening begins. This placement makes it difficult for the visiting bees to embrace all of the anthers at once, although it is commonly observed in other plant species having poricidal anthers buzzed by bees (Melo and Machado 1996; Fracasso and Sazima 2004; Oliveira-Rebouças and Gimenes 2004). The floral anatomy of *C. wurdackii* may favor cross-pollination as pollen is liberated from the lower anther group (indirectly, as a result of the buzzing of the upper group) and becomes attached to the end of the abdomen of most of the visitor bees where it is not easily transferred to the scopae and corbiculae. Additionally, herkogamy (which is a common characteristic in Melastomataceae flowers [Renner 1989]) causes the stigma to touch the visiting bee's abdomen before the anthers are buzzed, thus reducing the chances of self-pollination (Fracasso and Sazima 2004).

The flowers of *C. wurdackii* remain open for more than 24 hours and can be visited by diurnal, crepuscular, and nocturnal animals. In an investigation of pollination in *Silene alba* (Caryophyllaceae), Young (2002) observed that the flowers of this species remained open for more than 12 hours and were visited by animals with diurnal or nocturnal habits. The flowers of *C. wurdackii* were visited by bees with diurnal (*C. fuscata, B. atratus, X. cearensis, P*. cf. *callaina*, and *Euglossa* sp.) as well as crepuscular habits (*M. sodalis* and *P. dubia*), with the latter bee species being more frequent. The flowers of *C. hilariana* remained open for approximately 60 hours in the southern region of Brazil, but only received visits from diurnal bees (the genera *Centris, Bombus*, and *Xylocopa*), although this result was certainly influenced by the fact that their observations were limited to only the diurnal period (Fracasso and Sazima 2004).

The activities of the crepuscular bees (*M. sodalis* and *P. dubia*) in the present study appear to be influenced by light intensity. The numbers of visits of both bee species diminished greatly as light intensity increased, and a significant negative statistical correlation was observed between the number of visits and light intensity. The importance of light intensity to the daily activity of *Megalopta genalis* (a crepuscular bee) was also observed by Kelber et al. (2006) in Panama.

In addition to light intensity, temperature also appears to exercise an important role in the daily activity of crepuscular bee visitors to *C. wurdackii* flowers, as there was a significant negative statistical correlation between the temperature and floral visits. *P. dubia*, the largest and most pilose bee in the present study, was observed visiting *C. wurdackii* flowers more frequently than *M. sodalis* during periods of lower temperatures. The air temperature acts on the thermoregulation mechanisms of bees and other insects, but can be modified by their morphological characteristics (including size, number of bristles, and the color of their integument) (Herrera 1995). Additionally, some studies have established a relationship between buzzing behavior and the capacity of these bees to forage at low temperatures for the vibration of their flight muscles during buzzing helps increase their body temperature (Buchmann 1983; Renner 1989).

Large ocelli have been viewed as an essential morphological adaptation for bees foraging under low light (Kelber et al. 2006; Warrant et al. 2006; Warrant 2008; Somanathan et al. 2008). *P. dubia* and *M. sodalis* examined in the present study have both ocelli and head sizes typical of nocturnal and crepuscular bees (Kerfoot 1967). Ocelli have been shown to be the principal organs responsible for night vision in the Hymenoptera, while compound eyes are principally used during the day (Warrant et al. 2006). The strong relationship between the proportionally large size of their median ocelli and the capacity of *P. dubia* and *M. sodalis* to forage under conditions of low light intensity was likewise observed by studying other species of bees (Kerfoot 1967; Kelber et al. 2006), ants (Moser et al. 2004), and wasps (Warrant et al. 2006) with similar crepuscular and nocturnal habits.

In spite of the fact that all of the bee visitor species employed the same behavior patterns in removing pollen from the flowers of *C. wurdackii* (buzzing the anthers), not all of them could be considered potential pollinators. The smaller species (*P*. cf. *callaina* and *Euglossa* sp.) did not come into contact with the stigma during their floral visits, while the other diurnal species (*C. fuscata, B. atratus*, and *X. cearensis*) were observed successfully transferring pollen grains to the stigmas of these flowers. Nonetheless, considering the low numbers of visits of *C. fuscata, B. atratus*, and *X. cearensis* , they can only be considered occasional pollinators of *C. wurdackii*. On the other hand, in light of their morphological characteristics, the high frequency of their visits, and their behavior in the flower the crepuscular species, *M. sodalis* and *P. dubia*, were considered potential pollinators of *C. wurdackii*.

The floral anatomy of *C. wurdackii* indicates a melittophilous syndrome (Faegri and Van Der Pijl 1979). Although the yellow and orange colors of the flowers of *C. wurdackii* are considered to be typical attractants of diurnal bees, *C. fuscata, B. atratus*, and *X. cearensis* appeared to have only a small role in pollination in the present study. The flowers of *C. wurdackii* were visited more frequently by crepuscular bees, and these insects were also considered to be potential pollinators. In studying the pollination of *Parkia velutina* Benoist (Leguminosae: Mimosoideae) by nocturnal bees of the genus *Megalopta*, Hopkins et al. (2000) proposed that nocturnal melittophily in the genus *Parkia* represented an intermediate stage in the evolution of chiropterophily to entomophily within this genus, with the presence of characteristics of both syndromes (although the flowers also emitted a strong sweet odor that would preferentially attract insects). The flowers of *C. wurdackii* also produce a sweet odor that becomes stronger towards the end of the day. This characteristic is not common among the Melastomataceae and may represent an important attractant for the crepuscular bee species that pollinate these flowers.

The present study of pollination in *C. wurdackii* demonstrated that although these flowers have characteristics normally associated with the attraction of diurnal visitors, crepuscular visitors were actually potential pollinators. A number of workers have reported that plants that maintain their flowers open for more than 12 hours receive visits from nocturnal, crepuscular, and diurnal pollinators (Slauson 2000; Young 2002). Accordingly, studies of floral visitors to plants with flowers that remain open for long periods of time must necessarily include observations made during their entire receptive period.
